# Long-Term Administration of Angiotensin (1–7) to *db/db* Mice Reduces Oxidative Stress Damage in the Kidneys and Prevents Renal Dysfunction

**DOI:** 10.1155/2018/1841046

**Published:** 2018-10-23

**Authors:** Anna Malgorzata Papinska, Kathleen Elizabeth Rodgers

**Affiliations:** ^1^School of Pharmacy, University of Southern California, 1985 Zonal Ave., Los Angeles, CA 90033, USA; ^2^School of Medicine, Center for Innovation in Brain Sciences, University of Arizona, 1230 N. Cherry Ave., Tucson, AZ 85721, USA

## Abstract

**Aims:**

The goal of this study was to evaluate the effects of long-term (16 weeks) administration of angiotensin (1–7) [A(1–7)] on kidney function in *db/db* mice and to identify the protective mechanisms of this therapy.

**Methods:**

*db/db* mice and heterozygous controls were treated with A(1–7) or vehicle daily, subcutaneously for up to 16 weeks. Kidney injury was assessed by measuring blood flow in renal arteries, plasma creatinine levels, and proteinuria. Effects of treatment on oxidative stress were evaluated by histological staining and gene expression.

**Results:**

16 weeks of daily administration of A(1–7) to a mouse model of severe type 2 diabetes (*db/db*) prevented the progression of kidney damage. Treatment with A(1–7) improved blood flow in the renal arteries, as well as decreased plasma creatinine levels and proteinuria in diabetic mice. Reduction of oxidative stress was identified as one of the mechanisms of the renoprotective action of A(1–7). Treatment prevented formation of nitrotyrosine residues, a marker of oxidative stress damage. A(1–7) also reduced the expression of two enzymes involved in formation of nitrotyrosine, namely, eNOS and NOX-4. A(1–7) regulated the phosphorylation pattern of eNOS to enhance production of NO in diabetic animals, possibly through the Akt pathway. However, these elevated levels of NO did not result in increased nitrosylation, possibly due to reduced NOX-4 levels.

**Conclusions:**

Long-term administration of A(1–7) improved kidney function and reduced oxidative stress damage in *db/db* mice.

## 1. Introduction

Over 100,000 US citizens are diagnosed with kidney failure yearly and 44% of them are due to diabetes [1]. A central therapeutic strategy to prevent complications in patients with T2D is to reduce the activity of the pathological arm of the renin-angiotensin system (RAS), namely, levels and actions of angiotensin II (Ang-II). Ang-II has been implicated as a key factor in T2D pathology; however, the exact mechanism is not fully understood. Nonetheless, Ang-II has been shown to cause hypertension [2, 3], inflammation [4, 5], and oxidative stress [3, 6, 7] and contribute to insulin resistance [8, 9]. Thus, the current treatment for diabetic nephropathy focuses on angiotensin-converting enzyme inhibitors (ACEi), which reduce the production of Ang-II, and angiotensin receptor blockers (ARBs), which block the actions of Ang-II through its cognate receptor, AT1. However, both therapies are associated with adverse effects, are not safe to use during pregnancy [10, 11], and do not completely eliminate kidney damage. It is hypothesized that angiotensin (1–7) [A(1–7)], another member of the RAS, can ameliorate diabetic nephropathy without the side effects of ACEi or ARBs. A(1–7) has been shown to decrease hypertension and oppose pathological actions of Ang-II [12, 13]. Here, we demonstrate that long-term administration of A(1–7) to *db/db* mice, a model of severe T2D, acts renoprotective, at least in part, through the amelioration of oxidative stress.

Hyperglycemia, as well as high levels of cytokines and other growth factors, contributes to increased oxidative stress, which leads to numerous diabetic complications including nephropathy. Elevated levels of reactive oxygen species (ROS) may result in promotion of fibrosis, inflammation, and endothelial dysfunction in diabetic kidneys [14–17]. Studies show increased circulating levels of ROS in diabetes in both animals and patients [15, 18]. The main source of ROS in diabetic kidneys is thought to be NOX-4, a member of the NADPH oxidase family. Superoxide produced by this enzyme, when combined with nitric oxide (NO), results in the formation of peroxynitrite—a very potent ROS that has the capability to modify proteins to form nitrotyrosine (N-Tyr). Addition of the nitrate groups to the proteins changes their structure and function and may result in pathologies [19, 20].

Studies also show that A(1–7) [21] as well as its receptor Mas [22] are expressed in renal tissue in relatively high amounts and the peptide can be detected in urine [23]. This suggests that A(1–7) may play a role in kidney homeostasis. A(1–7) acts renoprotective in several models of kidney dysfunction [24, 25]. Studies of renal biopsies from patients with diabetic nephropathy also show an increased ACE/ACE2 ratio, which suggests increased production of Ang-II and decreased production of A(1–7). However, some studies show progression of kidney disease with A(1–7) treatment. For example, in streptozotocin-diabetic rats and in a nondiabetic model of unilateral ureteral obstruction [26], treatment with A(1–7) was not beneficial. These controversies have still not been fully explained; however, some of the discrepancies are being ascribed to dosage and route of administration. It is therefore crucial to further study the effects of A(1–7) on diabetic nephropathy to elucidate the mechanisms of possible protective action of this treatment.

## 2. Materials and Methods

### 2.1. Animal Procedures

We followed the methods of Papinska et al. [27].

All animal procedures were carried out in accordance with the Guide for the Care and Use of Laboratory Animals as adopted and promulgated by the US National Institutes of Health.

Eight-week-old male BKS.Cg-Dock7^m^ +/+ Lepr^db^/J (*db/db*) mice and age-matched heterozygous controls (nondiabetic) were purchased from Jackson Laboratories (Bar Harbor, ME, USA). Mice were randomized based on the initial body weight into four treatment groups (*n* = 6/group). Animals were kept on a 12 h light/dark cycle and food and water were available *ad libitum*.

Animals were administered either vehicle (saline) or A(1–7) (0.5 mg/kg/day) subcutaneously, daily for 16, 12, or 4 weeks. Previous dose finding studies performed in this laboratory revealed that 0.5 mg/kg/day, once daily, is the optimal dosing regimen, with no further benefit at 1 mg/kg/day [28, 29]. Pharmaceutical grade A(1–7) was purchased from Bachem (Torrance, CA, USA). At the necropsy, animals were overdosed with ketamine and xylazine cocktail followed by thoracotomy.

### 2.2. Ultrasonographic Assessment of Blood Flow Velocity in Renal Artery

Blood flow velocity in the renal arteries was assessed noninvasively using a high-frequency, high-resolution ultrasound system consisting of a Vivid 7 Dimension ultrasound machine equipped with a 6–13 MHz linear transducer (GE Healthcare, Little Chalfont, UK) after 16 weeks of treatment. Fur from the abdomen was removed using a hair removal cream. Anesthesia was induced with 3% isoflurane in an induction chamber. The mouse was then placed in a supine position on a heating pad to maintain body temperature at 36.5–37°C. Anesthesia was maintained through a nosecone and adjusted to maintain the heart rate at 450–550 beats per minute. The probe was positioned on the side of the abdomen to allow visualization of the cross section of the right kidney. Peak systolic blood flow velocity was measured using pulsed-wave Doppler in the renal artery just before it enters the kidney, as described previously [30]. Doppler measurements were than analyzed by a blinded observer.

### 2.3. Plasma and Urine Creatinine Concentration

Blood was collected from each animal at the necropsy through a cardiac puncture into EDTA-coated tubes. Immediately after collection, plasma was isolated by centrifugation and stored at −80°C until analysis. Urine samples were collected in the morning of the day preceding the necropsy. Creatinine concentration in the plasma and urine samples was measured using a Mouse Creatinine Kit (Crystal Chem, Downers Grove, IL) per the manufacturer's instructions.

### 2.4. Urine Protein Concentration

Protein concentration in urine samples was assessed using standard Bradford assay. BSA was used as a standard. The samples were incubated with Bradford reagent (Bio-Rad, Hercules, CA) for 10 min at room temperature and spectrophotometrically analyzed using a BioTek Synergy H1 Hybrid plate reader (BioTek, Winooski, VT). Protein concentration was normalized to urine creatinine levels.

### 2.5. Glomerular Hypertrophy and Mesangial Expansion

After 16 weeks of treatment, the kidneys were isolated, formalin-fixed, paraffin-embedded, and cut at 5 *μ*m. To assess glomerular hypertrophy and mesangial expansion, the sections were stained using the periodic acid-Schiff (PAS) method. Twenty images of random cortical glomeruli were obtained at 40x magnification. The images were analyzed in a blinded fashion using ImageJ (1.47v, NIH, USA). Glomerular area was measured using a free-hand selection tool, and staining was assessed using color-deconvolution plugin and threshold function. The mesangial expansion was expressed as percentage of glomerular area stained with PAS.

### 2.6. Immunohistochemistry for N-Tyr, p-eNOS, and NOX-4

Kidney sections were treated using a standard heat-induced antigen retrieval procedure. The slides were incubated with (a) rabbit polyclonal antibody directed against nitrated tyrosine residues (EMD Millipore, Billerica, MA) at 1 : 250 dilution, (b) rabbit polyclonal antibody to p-eNOS Ser1177 (GeneTex Inc., Irvine, CA) at 1 : 100 dilution, (c) rabbit polyclonal antibody to p-eNOS Thr495 (Bioss Inc., Woburn, MA) at 1 : 100 dilution, and (d) rabbit polyclonal antibody to NOX-4 (EMD Millipore, Billerica, MA) at 1 : 200 dilution. After incubation with a proper secondary antibody, an avidin-biotin complex method of detection was used. Twenty random images of renal cortex at 40x were evaluated for the extent of staining in a blinded fashion using ImageJ (1.47v, NIH, USA) and expressed as percentage of area positively stained.

### 2.7. Gene Expression

At the necropsy, left kidneys were isolated and stored in RNAlater (Thermo Fisher Scientific, Waltham, MA) at −20°C until use. qRT-PCR was performed as described previously [28]. Briefly, RNA was isolated using TRIzol reagent (Invitrogen by Thermo Fisher Scientific, Waltham, MA) and reverse-transcribed, and real-time PCR was performed using SYBR Green PCR Master Mix (Applied Biosystems by Life Technologies, Thermo Fisher Scientific, Waltham, MA). Relative expression of each of the genes of interest was evaluated using an ABI 7300 instrument (Applied Biosystems by Life Technologies, Thermo Fisher Scientific). Abundance of targeted mRNA was normalized against 29S mRNA.

### 2.8. Statistical Analysis

We followed the methods of Papinska et al. [27].

GraphPad Prism version 6.0c for Mac OS X (GraphPad Software, San Diego, CA, USA) was used to analyze the data. One-way ANOVA followed by Dunnett's multiple-comparison test were used to compare data. The level of statistical significance was set at 5%. Data are expressed as mean value ± standard error of the mean (SEM).

## 3. Results

### 3.1. 16 Weeks of A(1–7) Administration Reduces Shear Stress and Improves Kidney Function and Glomerular Structure in Diabetic Mice

**Figure 1:**
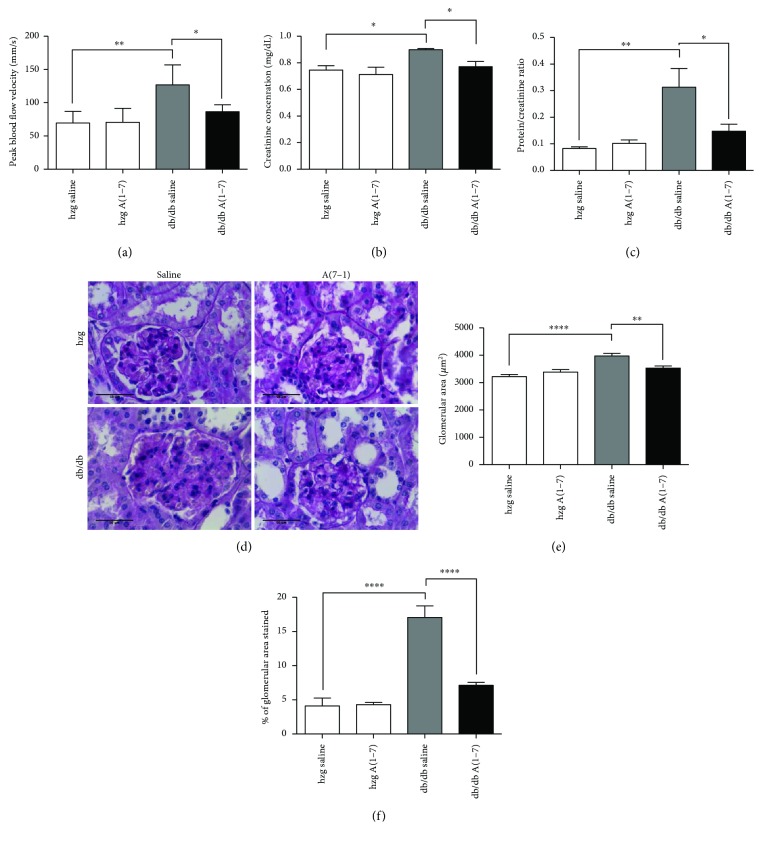
Physiological kidney function and glomerular structure in the animals treated for 16 weeks. The peak systolic blood velocity was increased in the renal arteries of diabetic animals. Treatment with A(1–7) reduced this measurement (a). Plasma creatinine and urine protein/creatinine ratio was assessed after 16 weeks of treatment to determine kidney function. Both parameters were increased in diabetic mice treated with saline and reduced after administration of A(1–7) (b, c). Glomerular health was assessed by measuring the glomerular area (e) and mesangial expansion (f). Mesangial expansion is expressed as percentage of glomerular area stained for extracellular matrix. Both glomerular hypertrophy and extent of fibrosis were increased in the diabetic animals treated with saline. Glomerular dysfunction was prevented by a 16-week treatment with A(1–7). Representative images of glomeruli stained using the periodic acid-Schiff method taken at 40x magnification are shown in (d). (hzg: heterozygous; *n* = 6 animals per group; ^∗^*p* < 0.05, ^∗∗^*p* < 0.01, and ^∗∗∗∗^*p* < 0.0001) Calculated using one-way ANOVA; plotted as mean with SEM.

The blood flow velocity in the renal artery was measured using ultrasonography. Treatment with A(1–7) reduced the blood flow velocity in renal arteries of diabetic mice (Figure 1(a)). *db/db* mice also had decreased kidney function as measured by creatinine levels in plasma and protein/creatinine ratio in urine (Figures 1(a) and 1(c)). Both of these parameters were decreased after treatment with A(1–7).

The glomerular structure was assessed by measuring glomerular hypertrophy and mesangial expansion. Both glomerular hypertrophy and the mesangial expansion were increased in kidneys from diabetic animals. A(1–7) reduced glomerular remodeling in *db/db* mice (Figure 1(d)–1(f)). No significant differences were seen between heterozygous (nondiabetic) groups [treated with A(1–7) or vehicle], which is also true for all the other parameters presented in this study.

### 3.2. Oxidative Stress Damage in Diabetic Kidneys Was Decreased after Administration of A(1–7)

**Figure 2:**
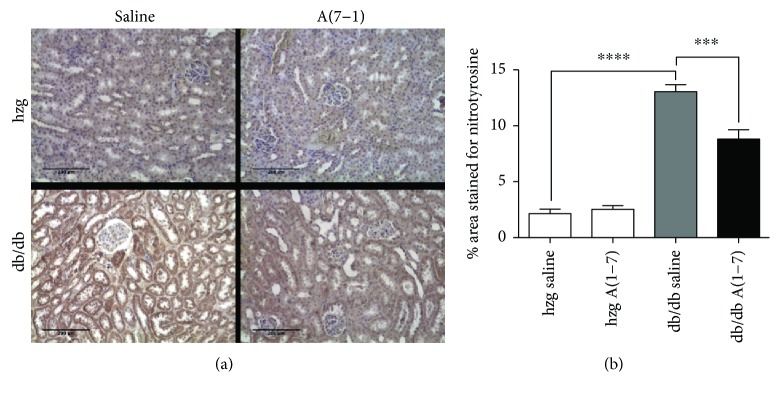
Oxidative stress damage in the kidneys from animals treated for 16 weeks. Damage due to oxidative stress was assessed using sections immunostained for nitrotyrosine residues. Representative images of kidney cortex taken at 10x magnification are shown in (a). Significantly, more staining was observed in the kidneys from diabetic animals treated with saline than in nondiabetic groups. 16 weeks of A(1–7) administration to *db/db* mice reduced oxidative stress damage in the kidneys (b). (hzg: heterozygous; *n* = 6 animals per group; ^∗∗∗^*p* < 0.001 and ^∗∗∗∗^*p* < 0.0001) Calculated using one-way ANOVA; plotted as mean with SEM.

NADPH oxidase, one of the main sources of superoxide anion in diabetic kidney, is directly activated by Ang-II. We evaluated oxidative stress-induced damage in the kidney sections using an anti-nitrotyrosine- (N-Tyr-) directed antibody. The extent of staining in the kidneys from diabetic mice treated with placebo was increased compared to nondiabetic animals (Figure 2). Administration of A(1–7) significantly reduced the presence of this marker, which suggests decreased oxidative stress damage in the kidneys.

### 3.3. Administration of A(1–7) Altered Gene Expression of eNOS and NADPH Oxidase

**Figure 3:**
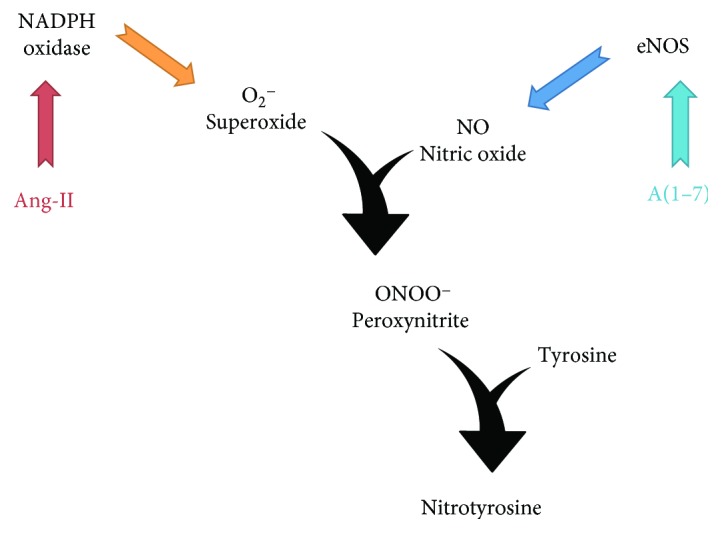
Formation of nitrotyrosine residues and involvement of eNOS and NADPH oxidase. Ang-II can directly activate NADPH oxidase to produce superoxide. A(1–7) is known to increase production of NO through activation of eNOS. Superoxide and NO react with each other to form peroxynitrite—a potent oxidant. Elevated levels of peroxynitrite cause nitration of proteins resulting in formation of nitrotyrosine residues that change the structure and function of proteins.

**Figure 4:**
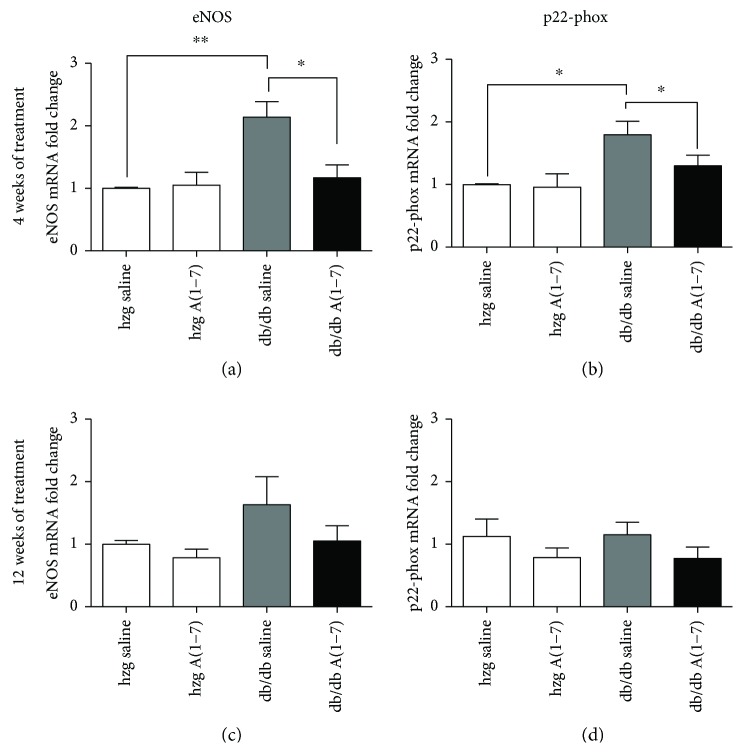
Gene expression of eNOS and NADPH oxidase in the kidneys from animals treated for 4 or 12 weeks. Gene expression of both eNOS and p22-phox (subunit of NADPH oxidase) was increased in diabetic animals after 4 weeks of treatment (animals were 12 weeks old) (a, b). Treatment with A(1–7) reduced the expression of both of these markers. No significant differences in gene expression of eNOS and p22-phox were detected in animals treated for 12 weeks (20 weeks old). (hzg: heterozygous; *n* = 6 animals per group; ^∗^*p* < 0.05 and ^∗∗^*p* < 0.01) Calculated using one-way ANOVA; plotted as mean with SEM.

eNOS is one of the main sources of NO in the kidneys. N-Tyr is formed due to increased levels of peroxynitrite, which is produced through reaction of NO with superoxide anion (Figure 3). We evaluated the expression of the two main enzymes that produce these molecules—eNOS and p22-phox (subunit of NADPH oxidase). Gene expression was performed on tissues collected from animals treated for either 4 or 12 weeks. Even though we did not observe any changes in the expression of these enzymes after 12 weeks of treatment (Figures 4(c) and 4(d)), there was a significantly increased expression of both eNOS and p22-phox in the kidneys of diabetic animals treated with the vehicle for 4 weeks compared to heterozygous mice (Figures 4(a) and 4(b)). Administration of A(1–7) reduced the gene expression of both of these enzymes in diabetic mice.

### 3.4. A(1–7) Alters the Phosphorylation Pattern of eNOS and Reduces Levels of NOX-4

**Figure 5:**
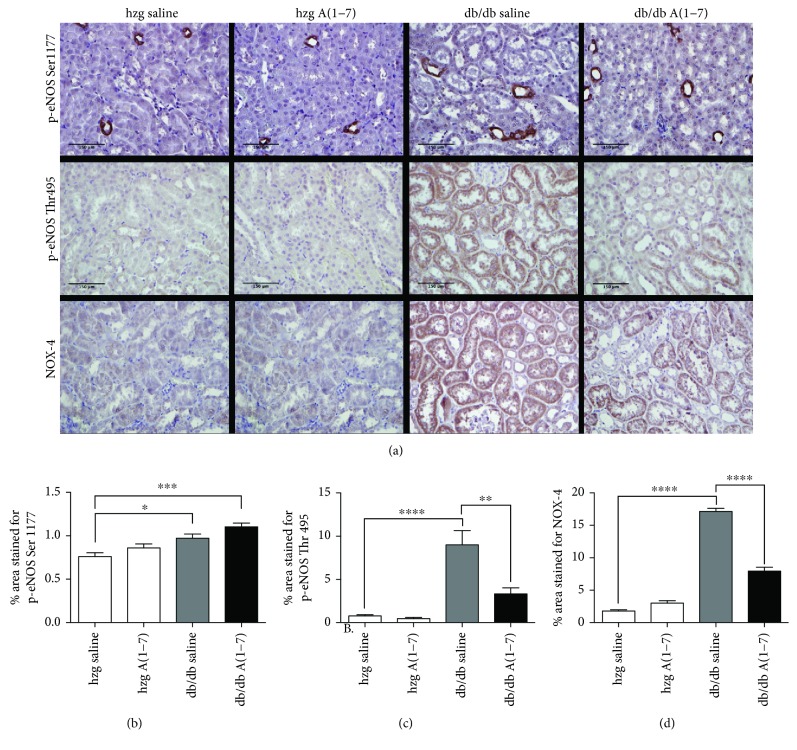
eNOS phosphorylation and NOX-4 expression in the kidneys from animals treated for 16 weeks. Levels of eNOS phosphorylated on Ser1177 (activating phosphorylation) and eNOS phosphorylated on Thr495 (deactivating phosphorylation), and NOX-4, a member of NADPH oxidase family, were assessed using immunohistochemistry in animals treated for 16 weeks. Representative images of kidney cortex taken at 40x magnification are shown in (a). Levels of both phosphorylation forms were increased in *db/db* mice from the control group. The extent of phosphorylation on Ser1177 was also increased in the diabetic animals treated with A(1–7) (b), whereas levels of phosphorylation on Thr495 were decreased in this group (c). The extent of staining for NOX-4 was increased in the diabetic animals from the control group compared to nondiabetic mice. Treatment with A(1–7) reduced the levels of NOX-4 in the kidneys of diabetic mice (d). (hzg: heterozygous; *n* = 6 animals per group; ^∗^*p* < 0.05, ^∗∗^*p* < 0.01, ^∗∗∗^*p* < 0.001, and ^∗∗∗∗^*p* < 0.0001) Calculated using one-way ANOVA; plotted as mean with SEM.

eNOS can be activated or deactivated through modification of the two main phosphorylation sites: phosphorylation of eNOS on Ser1177 and dephosphorylation on Thr495 activate eNOS to produce NO. We evaluated effects of A(1–7) on the eNOS phosphorylation pattern using immunohistochemistry. The phosphorylation on Ser1177 was increased in both *db/db* groups [treated with vehicle or A-(1–7)] (Figures 5(a) and 5(b)), whereas the phosphorylation on Thr495 was increased in diabetic animals treated with saline and reduced after treatment with A(1–7) (Figures 5(a) and 5(c)).

NOX-4 is the most prevalent form of NADPH oxidase in the kidneys and the main source of superoxide [31]. We evaluated the levels of NOX-4 in the kidney sections collected from animals treated for 16 weeks. Expression of NOX-4 was increased in the kidneys from diabetic animals treated with the vehicle as compared to heterozygous mice (Figures 5(a) and 5(d)). A(1–7) reduced the extent of staining for NOX-4 in *db/db* mice.

## 4. Discussion

Here, we show that the long-term administration of A(1–7) is effective in preventing oxidative stress damage and renal dysfunction in *db/db* mice. It is important to note that, so far, ACEi and ARBs even though able to slow down the progression of diabetic nephropathy are not recommended for prevention of kidney disease. As demonstrated previously, the treatment regimen presented in this article does not have any effects on blood glucose levels or body weight in this mouse model [27]. Thus, the effects of A(1–7) on the kidney's health occurred independently of blood glucose or obesity control.

In this study, A(1–7) reduced the blood flow velocity through the renal artery, which may have contributed to decreased shear stress in the kidneys and improved renal function. The increased levels of creatinine in plasma from diabetic animals treated with saline suggest impaired filtration function of the kidneys, whereas increased protein/creatinine ratio in the urine implies structural changes in the glomeruli. Treatment with A(1–7) reduced both of these parameters in the diabetic mice. Benter and colleagues showed similar results of Ang(1–7) treatment on renal function in streptozotocin-induced diabetes in rats [32, 33]. In this study, Ang(1–7) as well as its nonpeptide analog AVE0991 reduced proteinuria and improved vascular responsiveness of isolated ring segments from renal arteries. Mesangial expansion is another highlight of renal pathology in T2D. Mesangial cells produce excessive amounts of extracellular matrix, which reduces flexibility of the glomeruli and decreases surface area available for filtration. A(1–7) decreased mesangial expansion and glomerular hypertrophy, which might have contributed to enhancement of kidney function in diabetic animals.

Oxidative stress can be caused by both hyperglycemia and activation of the pathological arm of the RAS. Since peroxynitrite is one of the most potent oxidants, increased production of this molecule can lead to formation of N-Tyr and result in structural and functional changes to the proteins. Both NO and superoxide are needed to produce N-Tyr. We observed increased mRNA expression of both eNOS and p22-phox, a subunit of NADPH oxidase, in the kidneys from diabetic animals treated for 4 weeks but not in the kidneys from animals treated for 12 weeks. We hypothesize that the reason for this discrepancy is that the gene expression changes are an acute response to the stimuli. As anticipated, A(1–7) reduced the gene expression of both of these markers after 4 weeks of treatment, suggesting decreased production of both NO and superoxide at this time point. No significant changes after 16 weeks of treatment were anticipated, and therefore, this analysis was not performed.

The activation of eNOS is controlled through posttranslational changes such as phosphorylation. Increased phosphorylation of eNOS on Ser1177 in both diabetic groups suggests enhanced production of NO in these animals. We hypothesize that increased phosphorylation of eNOS on Thr495 observed in the diabetic group given the vehicle is associated with a defense mechanism that acts against the overproduction of ROS. In addition, phosphorylation on Ser1177 is known to be primarily associated with activation of the Akt pathway, which is involved in insulin signaling and is known to have a protective role in T2D [34, 35]. In contrast, activation of the PKC pathway, which is detrimental in diabetes [36], has been described as the main source of phosphorylation on Thr495. Increased phosphorylation on Thr495 in *db/db* mice from the placebo group may be therefore associated with increased activation of the PKC pathway. Further, reduction of the levels of phosphorylation at this amino acid suggests reduction in PKC activity in diabetic animals treated with A(1–7). High blood glucose is known to activate the PKC pathway, which is associated with overexpression of TGF-*β*, fibronectin, and collagen type IV; over time, this causes mesangial expansion [37].

In addition to enhanced activation of eNOS, kidneys from diabetic animals also showed increased expression of NOX-4, the most predominant type of NADPH oxidase in the kidneys, an enzyme responsible for cytosolic production of superoxide anion [31]. NADPH oxidase was initially discovered to be present in neutrophils and play a role in response to pathogens by producing high levels of ROS. In the kidneys, this enzyme is also located in nonphagocytic cell types such as mesangial cells, proximal tubules, vascular smooth muscle cells, endothelium, and fibroblasts [38]. It is thought that the primary role of ROS produced by the NADPH oxidase in the kidneys is to act as a secondary messenger. However, in pathological states, such as T2D, NADPH oxidase produces highly excessive amounts of ROS. Thus, NADPH oxidase contributes to oxidative stress damage in various renal pathologies including diabetic nephropathy [16, 39]. Overexpression of both p22-phox and NOX-4 subunits has been previously associated with diabetic nephropathy [40, 41]. In addition, inhibition of NADPH oxidase with apocynin was shown to reduce kidney damage and mesangial expansion in diabetic nephropathy [16, 40, 42]. In *db/db* mice, NOX-4 is involved in molecular mechanisms underlying renal fibrosis through increased TGF-beta and fibronectin production [38]. NADPH oxidase can also be activated via phosphorylation of PKC in diabetic kidneys [16]. Even though intracellular ROS may come from various sources, NOX-4 is thought to be a source of superoxide in the diabetic kidneys [31, 43]. NOX-4 has been recently shown to constitutively produce hydrogen peroxide in contrast to other forms of NOX that primarily produce superoxide; however, research showed that NOX-4 is in fact capable of releasing both hydrogen peroxide and superoxide [44]. Because treatment with A(1–7) reduced the levels of NOX-4 and decreased tyrosine nitration, we hypothesize that this is one of the major pathways that contribute to decreased oxidative stress damage in the kidneys. These findings are also consistent with work published by Benter and colleagues, who showed that A(1–7) administration to a rat model of streptozotocin-induced diabetes reduced activity of renal NADPH oxidase and decreased expression of NOX-4 [33]. Similarly, Dhaunsi et al. showed that Ang(1–7) can be effective in reducing NADPH oxidase activity in diabetic kidney [40].

Our findings are consistent with several recent studies in the field. Mori and colleagues showed that constant infusion of A(1–7) via implanted micro-osmotic pumps reduced renal hypertrophy and reduced mesangial expansion in older (5-6 months old) *db/db* mice [45]. The authors associated renoprotective effects of A(1–7) to reduction in oxidative stress, inflammation, fibrosis, and lipotoxicity. Giani and colleagues also demonstrated improved renal health in Zucker diabetic fatty rats treated with A(1–7) through a micro-osmotic pump for 2 weeks [46]. In this study, administration of Ang(1–7) reduced renal fibrosis, triglyceridemia, and proteinuria and improved creatinine clearance through reduction of blood pressure, oxidative stress, and inflammation.

Overall, A(1–7) may represent a novel, safer treatment for diabetic nephropathy. In contrast to ACEi and ARBs, A(1–7) activates the protective arm of the RAS. A(1–7) was shown to have positive effects on kidney cells even in the absence of a hemodynamic factor [47, 48]. Here, we confirm that long-term administration of A(1–7) in a severe model of T2D results in prevention of kidney damage and improved filtration function. One of the mechanisms involved in this renoprotective action is the amelioration of oxidative stress. We have also shown that administration of A(1–7) to healthy mice has virtually no effects on the kidneys. Clinical studies showed that A(1–7) is also safe in patients [49]. This allows for the rapid translation of our preclinical results into potential clinical evaluation.

## Data Availability

The data used to support the findings of this study are available from the corresponding author upon request.
